# Contribution of gene mutations to Silver-Russell syndrome phenotype: multigene sequencing analysis in 92 etiology-unknown patients

**DOI:** 10.1186/s13148-020-00865-x

**Published:** 2020-06-16

**Authors:** Takanobu Inoue, Akie Nakamura, Megumi Iwahashi-Odano, Kanako Tanase-Nakao, Keiko Matsubara, Junko Nishioka, Yoshihiro Maruo, Yukihiro Hasegawa, Hiroshi Suzumura, Seiji Sato, Yoshiyuki Kobayashi, Nobuyuki Murakami, Kazuhiko Nakabayashi, Kazuki Yamazawa, Tomoko Fuke, Satoshi Narumi, Akira Oka, Tsutomu Ogata, Maki Fukami, Masayo Kagami

**Affiliations:** 1 Department of Molecular Endocrinology, National Research Institute for Child Health and Development, 2-10-1 Okura, Setagaya-ku, Tokyo, 157-8535 Japan; 2 Department of Pediatrics, University of Tokyo, 7-3-1 Hongo, Bunkyo-ku, Tokyo, 113-8655 Japan; 3 Department of Pediatrics, Hokkaido University Graduate School of Medicine, Kita15, Nishi7, Kita-Ku, Sapporo, 060-8648 Japan; 4 Department of Pediatrics and Child Health, Kurume University School of Medicine, 67 Asahi-Machi, Kurume, 830-0011 Japan; 5 Department of Pediatrics, Shiga University of Medical Science, Seta Tsukinowa-cho, Otsu, 520-2192 Japan; 6 Division of Endocrinology and Metabolism, Tokyo Metropolitan Children’s Medical Center, 2-8-29 Musashidai, Fuchu, Tokyo, 183-8561 Japan; 7 Department of Pediatrics, Dokkyo Medical University, 880 Kitakobayashi, Mibu, 321-0293 Japan; 8 Department of Pediatrics, Saitama City Hospital, 2460, Mimuro, Midori-ku, Saitama, 336-8522 Japan; 9 Department of Pediatrics, Hiroshima University, 1-2-3 Kasumi, Minami-ku, Hiroshima, 734-8553 Japan; 10 Department of Pediatrics, Dokkyo Medical University Saitama Medical Center, 2-1-50, Minamikoshigaya, Koshigaya, 343-8555 Japan; 11 Department of Maternal-Fetal Biology, National Research Institute for Child Health and Development, 2-10-1 Okura, Setagaya-ku, Tokyo, 157-8535 Japan; 12 Medical Genetics Center, National Hospital Organization Tokyo Medical Center, 2-5-1 Higashigaoka, Meguro-ku, Tokyo, 152-8902 Japan; 13 Department of Pediatrics, Hamamatsu University School of Medicine, 1-20-1 Handayama, Higashi-ku, Hamamatsu, 431-3192 Japan

**Keywords:** Silver-Russell syndrome, Multigene sequencing, Functional analysis, *CDKN1C*, *PLAG1*, *IGF1R*, SHORT syndrome, Floating-Harbor syndrome, Pitt-Hopkins syndrome, Noonan syndrome

## Abstract

**Background:**

Silver-Russell syndrome (SRS) is characterized by growth failure and dysmorphic features. Major (epi)genetic causes of SRS are loss of methylation on chromosome 11p15 (11p15 LOM) and maternal uniparental disomy of chromosome 7 (upd(7)mat). However, *IGF2*, *CDKN1C*, *HMGA2*, and *PLAG1* mutations infrequently cause SRS. In addition, other imprinting disturbances, pathogenic copy number variations (PCNVs), and monogenic disorders sometimes lead to SRS phenotype. This study aimed to clarify the frequency and clinical features of the patients with gene mutations among etiology-unknown patients with SRS phenotype.

**Results:**

Multigene sequencing was performed in 92 out of 336 patients referred to us for genetic testing for SRS. The clinical features of the patients were evaluated based on the Netchine-Harbison clinical scoring system. None of the patients showed 11p15 LOM, upd(7)mat, abnormal methylation levels for six differentially methylated regions (DMRs), namely, *PLAGL1*:alt-TSS-DMR on chromosome 6, *KCNQ1OT1*:TSS-DMR on chromosome 11, *MEG3/DLK1*:IG-DMR on chromosome 14, *MEG3*:TSS-DMR on chromosome 14, *SNURF*:TSS*-*DMR on chromosome 15, and *GNAS A/B*:TSS-DMR on chromosome 20, PCNVs, or maternal uniparental disomy of chromosome 16. Using next-generation sequencing and Sanger sequencing, we screened four SRS-causative genes and 406 genes related to growth failure and/or skeletal dysplasia. We identified four pathogenic or likely pathogenic variants in responsible genes for SRS (4.3%: *IGF2* in two patients, *CDKN1C*, and *PLAG1*), and five pathogenic variants in causative genes for known genetic syndromes presenting with growth failure (5.4%: *IGF1R* abnormality (*IGF1R*), SHORT syndrome (*PIK3R1*), Floating-Harbor syndrome (*SRCAP*), Pitt-Hopkins syndrome (*TCF4*), and Noonan syndrome (*PTPN11*)). Functional analysis indicated the pathogenicity of the *CDKN1C* variant. The variants we detected in *CDKN1C* and *PLAG1* were the second and third variants leading to SRS, respectively. Our patients with *CDKN1C* and *PLAG1* variants showed similar phenotypes to previously reported patients. Furthermore, our data confirmed *IGF1R* abnormality, SHORT syndrome, and Floating-Harbor syndrome are differential diagnoses of SRS because of the shared phenotypes among these syndromes and SRS. On the other hand, the patients with pathogenic variants in causative genes for Pitt-Hopkins syndrome and Noonan syndrome were atypical of these syndromes and showed partial clinical features of SRS.

**Conclusions:**

We identified nine patients (9.8%) with pathogenic or likely pathogenic variants out of 92 etiology-unknown patients with SRS phenotype. This study expands the molecular spectrum of SRS phenotype.

## Background

Silver-Russell syndrome (SRS) is a clinically and genetically heterogeneous disorder characterized by growth failure and dysmorphic features [1]. Recently, the Netchine-Harbison clinical scoring system (NH-CSS) has been adopted as the clinical diagnostic criteria of SRS [1]. NH-CSS has the following six key features: (1) small for gestational age (SGA), (2) postnatal growth failure, (3) relative macrocephaly at birth, (4) protruding forehead, (5) body asymmetry, and (6) feeding difficulties and/or low body mass index [1]. The patients with four or more NH-CSS criteria have a diagnosis of SRS [1]. Among these patients, patients satisfying NH-CSS criteria including both relative macrocephaly and protruding forehead, but with normal molecular testing, are classified as “clinical SRS” [1]. In addition, many patients with SRS show clinical features such as triangular face, fifth finger clinodactyly, and/or brachydactyly [2]. Patients meeting only three NH-CSS criteria, but who have clinically suspected SRS were recommended to receive genetic testing for SRS as well as patients with four or more NH-CSS criteria [1].

The major (epi)genetic causes of SRS are loss of methylation on chromosome 11p15 (11p15 LOM) and maternal uniparental disomy of chromosome 7 (upd(7)mat) [1]. Among patients without 11p15 LOM or upd(7)mat, other imprinting disturbances such as Temple syndrome, maternal uniparental disomy of chromosome 16 (upd(16)mat), maternal uniparental disomy of chromosome 20, pathogenic copy number variations (PCNVs), and mutations in *IGF2* on the paternal allele and *CDKN1C* on the maternal allele, which are causative genes for SRS, were identified in some cases [1]. Recently, *HMGA2* on 12q14 and *PLAG1* on 8q12 were proposed as the new responsible genes for SRS [3, 4]. Patients with mutations of these SRS-causative genes have a risk of transmitting the disorder [1, 3, 4]. In addition, some monogenic disorders such as 3-M syndrome, Mulibrey nanism, SHORT syndrome, Floating-Harbor syndrome, and IMAGe syndrome are recognized as differential diagnoses of SRS [1].

To clarify the frequency and clinical features of the patients with gene mutations among etiology-unknown patients with SRS phenotype, we performed multigene sequencing for four SRS-causative genes and 406 genes related to growth failure and/or skeletal dysplasia in 92 patients with SRS phenotype who did not have 11p15 LOM, upd(7)mat, other imprinting disturbances, or PCNVs.

## Results

### Molecular analysis

**Fig. 1:**
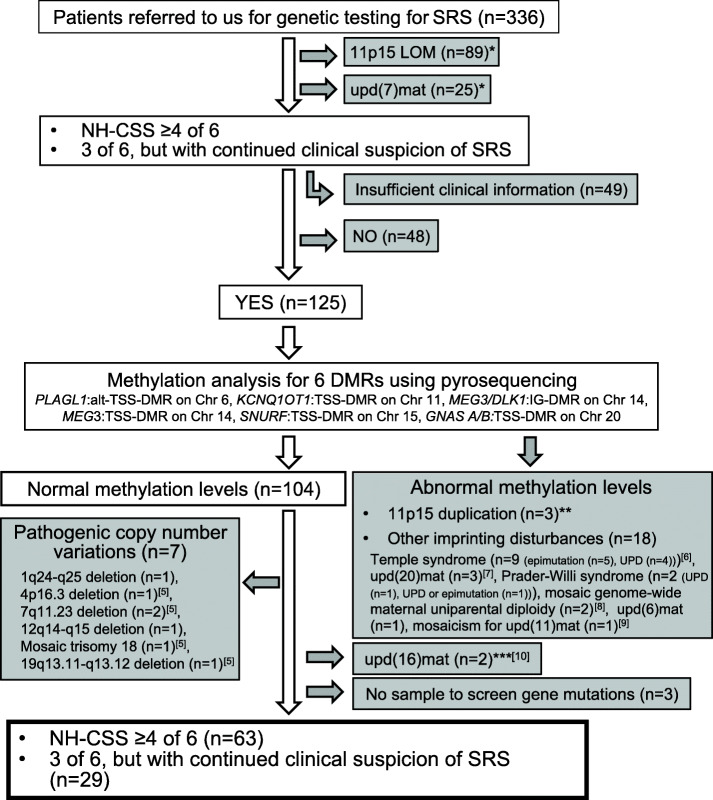
Flowchart of inclusion criteria. A total of 336 patients were referred to us for genetic testing for Silver-Russell syndrome (SRS) from 2002 to 2018. Our study included 92 patients without pathogenic copy number variations or abnormal methylation levels for ten differentially methylated regions (DMRs), namely, *H19/IGF2*:IG-DMR, *PEG10*:TSS-DMR, *MEST*:alt-TSS-DMR, *PLAGL1*:alt-TSS-DMR, *KCNQ1OT1*:TSS-DMR, *MEG3/DLK1*:IG-DMR, *MEG3*:TSS-DMR, *SNURF*:TSS*-*DMR, *ZNF597*:TSS-DMR, and *GNAS A/B*:TSS-DMR. 11p15 LOM, loss of methylation on chromosome 11p15; upd(7)mat, maternal uniparental disomy of chromosome 7; NH-CSS, Netchine-Harbison clinical scoring system; Chr, chromosome; upd(20)mat, maternal uniparental disomy of chromosome 20; upd(6)mat*,* maternal uniparental disomy of chromosome 6; upd(11)mat*,* maternal uniparental disomy of chromosome 11; upd(16)mat, maternal uniparental disomy of chromosome 16. *We evaluated clinical features of only a part of the patients according to the Netchine-Harbison clinical scoring system. **The duplicated region of two patients with 11p15 duplications did not include the *H19/IGF2*:IG-DMR. Thus, these patients showed normal methylation levels of the *H19/IGF2*:IG-DMR. The duplicated region of the remaining one patient included the *H19/IGF2*:IG-DMR. The methylation level of the *H19/IGF2*:IG-DMR in this patient was low normal, and we did not recognize 11p15 LOM. ***We began upd(16)mat screening in 2016. As such, we performed upd(16)mat screening for only a part of the patients with pathogenic copy number variations and patients with abnormal methylation levels of the DMRs related to known imprinting disorders before 2016

We analyzed 92 SRS phenotypic patients out of 336 patients referred to us for genetic testing for SRS. The clinical features of the patients were evaluated based on the Netchine-Harbison clinical scoring system. None of the patients had 11p15 LOM, upd(7)mat, abnormal methylation levels for six differentially methylated regions (DMRs), namely, *PLAGL1*:alt-TSS-DMR on chromosome 6, *KCNQ1OT1*:TSS-DMR on chromosome 11, *MEG3/DLK1*:IG-DMR on chromosome 14, *MEG3*:TSS-DMR on chromosome 14, *SNURF*:TSS*-*DMR on chromosome 15, and *GNAS A/B*:TSS-DMR on chromosome 20, PCNVs, or upd(16)mat (Fig. 1) [5–10]. We performed multigene screening for four genes responsible for SRS and 406 genes related to growth failure and/or skeletal dysplasia (Additional file 1: Table S1). All rare variants were evaluated based on the American College of Medical Genetics Standards and Guidelines [11]. We extracted the variants classified as “pathogenic” or “likely pathogenic.” We detected nine patients (9.8%) with pathogenic or likely pathogenic variants out of 92 etiology-unknown patients with SRS phenotype. Four variants were in responsible genes for SRS (4.3%: *IGF2* in two patients, *CDKN1C*, and *PLAG1*) and five variants were in causative genes for known genetic syndromes presenting with growth failure (5.4%: *IGF1R* abnormality (*IGF1R*), SHORT syndrome (*PIK3R1*), Floating-Harbor syndrome (*SRCAP*), Pitt-Hopkins syndrome (*TCF4*), and Noonan syndrome (*PTPN11*)) (Table 1 and Additional file 2: Figure S1).

**Table 1:** Pathogenic or likely pathogenic variants detected in this study

Patient	Pathogenic or likely pathogenic variants in the responsible genes for SRS				Pathogenic variants in causative genes for known genetic syndromes presenting with growth failure				
	Patient 1 [12]	Patient 2 [12]	Patient 3	Patient 4	Patient 5	Patient 6	Patient 7	Patient 8	Patient 9
Gene	*IGF2*	*IGF2*	*CDKN1C*	*PLAG1*	*IGF1R*	*PIK3R1*	*SRCAP*	*TCF4*	*PTPN11*
Variant	c.209G>A	c.211T>C	c.947G>A	c.589C>T	c.1457delC	c.1892G>A	c.7376delC	c.1102_1103delCA	c.844A>G
	p.(Cys70Tyr)	p.(Cys71Arg)	p.(Arg316Gln)	p.(Arg197*)	p.(Ser487Profs*21)	p.(Arg631Gln)	p.(Pro2459Leufs*16)	p.(Gln368Glyfs*6)	p.(Ile282Val)
Genetic diagnosis	SRS	SRS	SRS	SRS	*IGF1R* abnormality	SHORT syndrome	Floating-Harbor syndrome	Pitt-Hopkins syndrome	Noonan syndrome
Inheritance	De novo	De novo or paternal	Mother (carrier)	Mother (affected)	Father (carrier)	De novo	De novo	De novo	De novo
Allele	Paternal	Paternal	Maternal	Maternal	Paternal	NE	NE	NE	NE
Karyotype	46,XY	46,XY	46,XX	46,XX	NE	46,XY	46,XY	46,XX	46,XY
Allele frequency									
gnomAD [13]	None	None	None	None	None	None	None	None	None
HGVD [14]	None	None	None	None	None	None	None	None	None
4.7KJPN [15]	None	None	None	None	None	None	None	None	None
In silico pathogenicity prediction									
CADD [16]	1% most deleterious	1% most deleterious	1% most deleterious	1% most deleterious	1% most deleterious	1% most deleterious	1% most deleterious	1% most deleterious	1% most deleterious
(PHRED score)	27.4	27.2	32.0	37.0	35.0	33.0	27.2	35.0	23.6
MutationTaster [17] (score)	Disease causing	Disease causing	Disease causing	Disease causing	Disease causing	Disease causing	Disease causing	Disease causing	Disease causing
	1.000	1.000	0.662	1.000	1.000	1.000	1.000	1.000	1.000
SIFT [18] (score)	Damaging	Damaging	Damaging	–	–	Damaging	–	–	Damaging
	0.000	0.000	0.000			0.000			0.000
PP2_HVAR [19] (score)	Probably damaging	Probably damaging	Probably damaging	–	–	Probably damaging	–	–	Benign
	0.975	0.933	0.982			0.941			0.088
M-CAP [20] (score)	Possibly pathogenic	Possibly pathogenic	Possibly pathogenic	–	–	Possible pathogenic	–	–	Possibly pathogenic
	0.867	0.887	0.964			0.568			0.033
ACMG classification [11] criteria	Pathogenic	Likely pathogenic	Likely pathogenic	Pathogenic	Pathogenic	Pathogenic	Pathogenic	Pathogenic	Pathogenic
	PS2, PM1, PM2, PP3, PP4	PM1, PM2, PP3, PP4	PS3, PM2, PM5^a^, PP3	PVS1, PM2, PP1, PP3	PVS1, PM2, PP3	PS1, PS2, PM2, PP3	PSV1, PS1, PS2, PM2, PP3	PSV1, PS2, PM2, PP3, PP4	PS1, PS2, PM2

Accession number *IGF2* NM_000612.6, *CDKN1C* NM_000076.2, *PLAG1* NM_002655.3, *IGF1R* NM_000875.5, *PIK3R1* NM_181523.3, *SRCAP* NM_006662.3, *TCF4* NM_001083962.2, and *PTPN11* NM_002834.5

*SRS* Silver-Russell syndrome*, NE* not examined, *gnomAD* Genome Aggregation Database, *HGVD* Human Genetic Variation Database, *4.7KJPN* allele and genotype frequency panel from 4.7 K Japanese individuals, *CADD* Combined Annotation Dependent Depletion, *SIFT* Sorting Intolerant From Tolerant, *PP2* Polymorphism Phenotyping v2, *M-CAP* Mendelian Clinically Applicable Pathogenicity

^a^A different missense variant (p.(Arg316Trp)) was reported in a patient with Bechwith-Wiedemann syndrome [21]

**Fig. 2:**
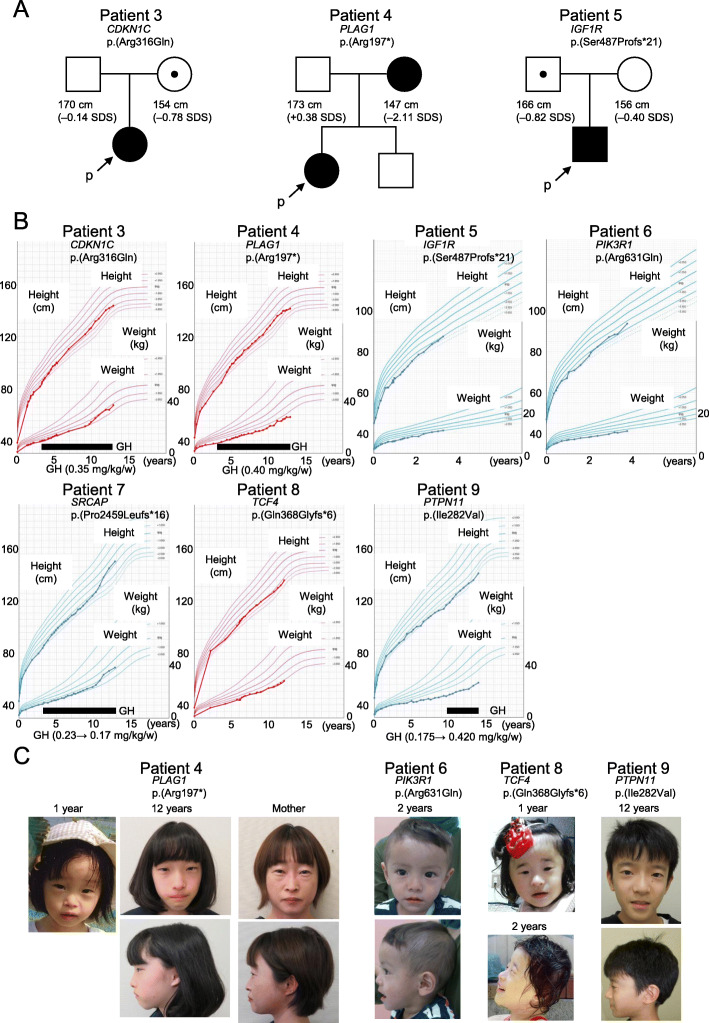
Clinical findings of the patients identified in this study. **a** Pedigrees of patients with variants inherited from their parents. **b** Growth charts. **c** Photographs of the patients and the mother of patient 4. Patients 1 and 2 were already reported [12]. SDS, standard deviation score; GH, growth hormone

Patients 1 and 2 with *IGF2* variants were already reported [12]. Both two variants, p.(Cys70Tyr) and p.(Cys71Arg), were predicted to disrupt S-S bindings in the IGF2 protein [22]. Patient 3 showed a rare variant, p.(Arg316Gln), causing amino acid alteration at the C-terminal of CDKN1C protein in the PCNA-binding domain[23]. This variant was inherited from her mother with normal height (Fig. 2a). To confirm pathogenicity of this variant, we performed functional analysis. Patient 4 showed a nonsense variant, p.(Arg197*), in *PLAG1*. Her mother with severe short stature also had the same variant (Fig. 2a). Patient 5 demonstrated a pathogenic frameshift variant, p.(Ser487Profs*21), in *IGF1R*. His father without severe short stature also showed the same variant (Fig. 2a). Patient 6 showed a missense variant, p.(Arg631Gln), in *PIK3R1*. This variant was previously reported in the patient with SHORT syndrome [24]. Patient 7 showed a frameshift variant, p.(Pro2459Leufs*16), in *SRCAP*, which is the causative gene for Floating-Harbor syndrome[25]. Patient 8 had a novel frameshift variant, p.(Gln368Glyfs*6), in *TCF4*. *TCF4* is a causative gene for Pitt-Hopkins syndrome [26]. Patient 9 had a missense variant, p.(Ile282Val), in *PTPN11*, which was previously reported as a pathogenic gene mutation for Noonan syndrome [27]. Both parents of patients 6, 7, 8, and 9 did not have the variants identified in their children.

### Functional analysis

**Fig. 3:**
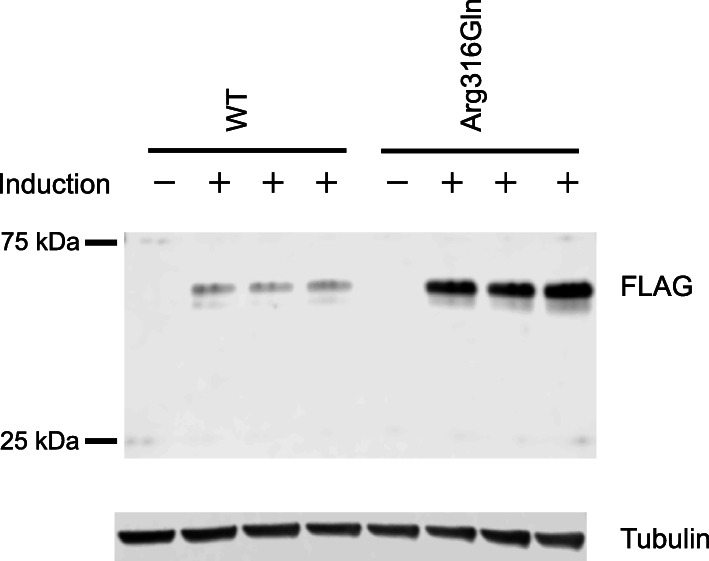
Results of Western blot analysis. The doxycycline-inducible protein expression level of Arg316Gln-CDKN1C was higher than that of WT-CDKN1C. The experiment was conducted in triplicate. WT, wildtype

To evaluate the effect of the identified *CDKN1C* variant (Arg316Gln) on protein expression, we created expression vectors encoding the CDKN1C protein, expressed each CDKN1C protein, and detected them with Western blotting. The analysis revealed that the protein expression level of Arg316Gln-CDKN1C was higher than that of wildtype (WT)-CDKN1C (Fig. 3), suggesting that the variant positively affected the stability of CDKN1C protein.

### Clinical analysis

Clinical features of the nine patients with pathogenic or likely pathogenic variants are summarized in Table 2. All four patients with variants in the genes responsible for SRS (*IGF2*, *CDKN1C*, and *PLAG1*) had a diagnosis of “clinical SRS.” However, only two out of five patients with variants in causative genes for known genetic syndromes had a diagnosis of “clinical SRS.” Patients 1 and 2 with *IGF2* variants showed motor and intellectual developmental delay, genital features, and high NH-CSS score (≥ 5/6) including feeding difficulties[12]. Patient 3 with a *CDKN1C* variant satisfied four NH-CSS criteria, but she did not show body asymmetry. She did not show other clinical features such as adrenal insufficiency or metaphyseal dysplasia, which were observed in the patients with IMAGe syndrome with gene mutation in the PCNA-binding domain of *CDKN1C* [23]. Growth hormone (GH) therapy was performed from 3 to 12 years old and was effective for her height (Fig. 2b). Her intellectual development was normal. Patient 4 with a *PLAG1* variant met NH-CSS criteria, including protruding forehead (Fig. 2c), but she did not show body asymmetry. She demonstrated severe feeding difficulties requiring cyproheptadine for appetite stimulation and enteral nutrient. Although she had diagnosed attention-deficit hyperactivity disorder (ADHD) and was not good at her studies, she showed apparently normal intellectual development and went to a regular class at school. After GH therapy was started, she developed hypothyroidism and required levothyroxine. Her father and brother were within normal height; however, her mother with the same variant was 147 cm tall (− 2.11 standard deviation score (SDS)) (Fig. 2a). She was born at term with the weight of 2150 g. She did not have typical facial features of SRS, such as protruding forehead and triangular face in adulthood (Fig. 2c). Unfortunately, her photograph at infancy was not available.

**Table 2:** Clinical features of the patients with pathogenic or likely pathogenic variants

Patient	Patients with pathogenic or likely pathogenic variants in the responsible genes for SRS				Patients with pathogenic variants in causative genes for known genetic syndromes presenting with growth failure				
	Patient 1 [12]	Patient 2 [12]	Patient 3	Patient 4	Patient 5	Patient 6	Patient 7	Patient 8	Patient 9
Genetic diagnosis	SRS	SRS	SRS	SRS	*IGF1R* abnormality	SHORT syndrome	Floating-Harbor syndrome	Pitt-Hopkins syndrome	Noonan syndrome
Gene	*IGF2*	*IGF2*	*CDKN1C*	*PLAG1*	*IGF1R*	*PIK3R1*	*SRCAP*	*TCF4*	*PTPN11*
Variant	p.(Cys70Tyr)	p.(Cys71Arg)	p.(Arg316Gln)	p.(Arg197*)	p.(Ser487Profs*21)	p.(Arg631Gln)	p.(Pro2459Leufs*16)	p.(Gln368Glyfs*6)	p.(Ile282Val)
Sex	Male	Male	Female	Female	Male	Male	Male	Female	Male
Age at the last examination (years)	8	15	12	12	3	3	12	12	13
Gestational age (weeks:days)	36:0	37:4	33:1	38:5	39:3	41:1	38:2	36:0	33:6
Birth length in cm (SDS)^a^	34.5 (− 3.85)	39.0 (− 3.54)	38.3 (− 1.95)	42.0 (− 3.12)	45.0 (− 2.11)	47.0 (− 1.65)	43.0 (− 2.49)	38.0 (− 2.97)	45.0 (+ 0.47)
Birth weight in g (SDS)^a^	1164 (− 4.23)	1336 (− 4.23)	1120 (− 2.85)	1804 (− 3.28)	2292 (− 2.22)	2386 (− 3.01)	2332 (− 1.90)	1508 (− 2.79)	2130 (+ 0.04)
Birth OFC in cm (SDS)^a^	31.0 (− 0.72)	30.8 (− 1.49)	28.1 (− 1.12)	32.0 (− 0.86)	32.9 (− 0.28)	33.0 (− 0.54)	32.0 (− 0.80)	28.5 (− 2.28)	31.8 (+ 0.68)
Height at 24 months in cm (SDS)^b,c^	70.9 (− 4.63)	72.5 (− 4.29)	75.1 (− 3.34)	77.6 (− 3.42)	78.9 (− 2.38)	77.9 (− 2.50)	79.2 (− 3.37)	81.4 (− 1.44)	80.7 (− 2.20)
BMI at 24 months (SDS)^b,c^	− 2.70	− 1.19	− 1.26	− 2.76	− 2.09	− 1.94	0.44	− 3.19	− 1.29
Height at the last examination in cm (SDS)^b^	108.8 (− 3.26)	141.5 (− 4.68)	143.5 (− 1.73)	141.6 (− 2.06)	87.9 (− 2.03)	93.8 (− 1.27)	149.2 (− 0.76)	134.9 (− 2.33)	140.1 (− 3.09)
Weight at the last examination in kg (SDS)^b^	14.6 (− 5.25)	30.5 (− 5.81)	36.9 (− 1.22)	28.0 (− 2.99)	11.5 (− 1.79)	11.2 (− 2.70)	38.6 (− 0.85)	28.1 (− 2.17)	26.6 (− 4.31)
GH treatment	6 years~	7 ~ 15 years	3 ~ 12 years	3 ~ 12 years	−	−	3 years~	−	9 years~
SGA^d^	+	+	+	+	+	+	+	+	−
Postnatal growth failure^c,e^	+	+	+	+	+	+	+	−	+
Relative macrocephaly at birth^f^	+	+	+	+	+	+	+	−	−
Protruding forehead	+	+	+	+	+	+	−	+	+
Body asymmetry	−	+	−	−	−	−	−	−	−
Feeding difficulties and/or low BMI	+	+	−	+	+	−	−	+	+
NH-CSS	5/6	6/6	4/6	5/6	5/6	4/6	3/6	3/6	3/6
Triangular face	+	+	+	+	+	+	+	−	+
Fifth finger clinodactyly	+	+	−	+	+	−	+	+	−
Fifth finger brachydactyly	+	+	−	+	+	−	+	+	+
Present characteristic features of genetic syndrome caused by the pathogenic variant					SGA, postnatal growth failure, feeding difficulties, triangular face, high plasma IGF-1 levels	SGA, postnatal growth failure, protruding forehead, triangular face, micrognathia, dental delay, low-set ears	SGA, postnatal growth failure, relative macrocephaly, triangular face, delayed bone age in early childhood, speech delay, bulbous nose, broad fingertips	Narrow forehead, thin lateral eyebrows, wide nasal bridge/ridge/tip, flared nasal alae, full cheeks/prominent midface, wide mouth/full lips/cupid bow upper lip, thickened/overfolded helices, severe developmental delay, breathing regulation anomalies (intermittent hyperventilation, apnea) (10 years~)	Postnatal growth failure, triangular face, chest deformity, cubitus valgus, hypertelorism
Absent characteristic features of genetic syndrome caused by the pathogenic variant					Microcephaly, pectus excavatum, developmental delay	Lipoatrophy, inguinal hernia, deep-set eyes, Rieger abnormality, hyperextensibility of joints, speech delay, sensorineural deafness	Long eyelashes, deep-set eyes, short philtrum	Myopia, constipation, unstable gait	Pulmonary valve stenosis, hypertrophic cardiomyopathy, lymphedema, disordered bleeding, cryptorchidism, down-slanting of palpebral fissures, ptosis, low-set ears, low posterior hairline, webbed neck
Development									
Motor developmental delay	+	+	−	−	−	−	−	+	−
Age at head control (months)	5	6	4	4	4	4	Unknown	12	4
Age at sitting without support (years)	1.0	0.8	0.6	0.7	0.5	0.7	Unknown	Unknown	0.8
Age at walking without support (years)	1.5	1.3	1.0	1.0	1.3	1.3	Unknown	4.8	1.1
Speech delay	+	+	−	−	−	−	+	+	−
IQ/DQ (age at examination)	77 (3 years)	79 (6 years)	Unknown	Unknown	NE	NE	Unknown	17 (6 years)	NE
Other features	Cleft palate, hypospadias, bifid scrotum, hypoplastic finger nails	Cleft palate, hypospadias, syndactyly	Left inguinal hernia, café au lait spot, Mongolian spot, bronchial asthma, dysmenorrhea	ADHD, hypothyroidism (5 years~)	−	−	Perthes disease, hematoma in the left temporal lobe, atopic dermatitis	−	−
Paternal height in cm (SDS)	Unknown	169 (− 0.31)	170 (− 0.14)	173 (+ 0.38)	166 (− 0.82)	170 (− 0.14)	174 (+ 0.55)	170 (− 0.14)	187 (+ 2.78)
Maternal height in cm (SDS)	150 (− 1.54)	159 (+ 0.17)	154 (− 0.78)	147 (− 2.11)	156 (− 0.40)	158 (− 0.02)	161 (+ 0.55)	156 (− 0.40)	167 (+ 1.70)

*SRS* Silver-Russell syndrome, *SDS* standard deviation score, *OFC* occipitofrontal circumference, *BMI* body mass index, *GH* growth hormone, *SGA* small for gestational age, *NH-CSS* Netchine-Harbison clinical scoring system, *IQ* intelligence quotient, *DQ* developmental quotient, *NE* not examined, *ADHD* attention-deficit hyperactivity disorder

^a^Birth length, weight, and OFC were evaluated by the sex- and the gestational age-matched Japanese reference data (http://jspe.umin.jp/medical/chart_dl.html)

^b^ Postnatal height, BMI, and weight were evaluated by the sex- and the age-matched Japanese reference data (http://jspe.umin.jp/medical/chart_dl.html)

^c^If we did not get information at 24 ± 1 months, we used the data at the nearest measure available older than 25 months

^d^Birth length and/or birth weight ≤ − 2 SDS

^e^Height at 24 ± 1 months ≤ − 2 SDS or height ≤ − 2 SDS below mid-parental target height. Mid-parental target height was calculated as follows: ((father’s height + mother’s height)/2) + 6.5 cm for boys and − 6.5 cm for girls.

^f^Head circumference at birth ≥ 1.5 SDS above birth length and/or weight SDS

Patients 5, 6, and 7 had genetically diagnosed *IGF1R* abnormality, SHORT syndrome, and Floating-Harbor syndrome, respectively, which have phenotypic overlap with SRS [1]. Patient 5 with five NH-CSS criteria showed many clinical features observed in the patients with *IGF1R* abnormality, such as growth failure and triangular face [28]. His plasma IGF-1 level was 121 ng/mL (+ 1.36 SDS) at 1 year old [29]. The father of patient 5 with the same *IGF1R* abnormality was 166 cm tall (− 0.82 SDS) and was born appropriate for gestational age (Fig. 2a). Patient 6 satisfying NH-CSS criteria had many symptoms of SHORT syndrome, such as prenatal and postnatal growth failure, characteristic facial features, and dental delay, but he did not show lipoatrophy nor hyperextensibility of joints (Fig. 2c) [24]. Patient 7 had three NH-CSS criteria and triangular face, fifth finger clinodactyly, and brachydactyly, together with Floating-Harbor syndrome-like clinical features, such as delayed bone age in his childhood, speech delay, bulbous nose, and broad fingertips [25]. GH treatment for SGA-short stature was started at 3 years old. During GH treatment, the dose was reduced due to development of Perthes disease (Fig. 2b). Hematoma in the left temporal lobe was incidentally detected by head magnetic resonance imaging. In addition, he suffered from severe atopic dermatitis.

Patients 8 and 9 had genetically diagnosed Pitt-Hopkins syndrome and Noonan syndrome, respectively, which are associated with growth failure [26, 30]. Patient 8 showed three NH-CSS criteria, including protruding forehead with fifth finger clinodactyly and brachydactyly together with severe developmental delay, but not breathing regulation anomalies characteristic of Pitt-Hopkins syndrome [26], when she was referred to us for genetic examination (Fig. 2c). Patient 9 had three NH-CSS criteria and triangular face and fifth finger brachydactyly together with only partial clinical features of Noonan syndrome, such as chest deformity, cubitus valgus, and hypertelorism (Fig. 2c) [30].

## Discussion

Out of the 92 patients with SRS phenotype, we identified four patients (4.3%) with pathogenic or likely pathogenic variants in responsible genes for SRS, and five patients (5.4%) with pathogenic variants in causative genes for known genetic syndromes showing growth failure. To our knowledge, three studies about multigene screening of etiology-unknown patients with SRS phenotype using next-generation sequencing have been reported (Additional file 3: Table S2) [3, 31, 32]. The numbers and clinical features of the patients, target genes, methods of multigene sequencing, and molecular analyses before multigene sequencing are different among these studies. Our study is the largest multigene sequencing study to date, including only the patients evaluated based on NH-CSS, and showed that around 10% of etiology-unknown patients with SRS phenotype are caused by pathogenic or likely pathogenic variants of the causative genes for SRS or genetic syndromes related to growth failure. In addition, our study included a single familial case and previous studies also reported two familial cases (Additional file 3: Table S2) [3, 31]. Because onsets in almost all of the patients with 11p15 LOM and upd(7)mat are sporadic [1], family history in patients with SRS phenotype suggests other mechanisms of their etiologies.

In this study, we identified patients 1 and 2 with *IGF2* variants, which were previously reported [12], and patient 3 with a *CDKN1C* variant. This variant detected in patient 3 is the second variant in *CDKN1C* leading to SRS. CDKN1C protein inhibits cell growth [23]. Consistent with this, loss-of-function mutations result in Beckwith-Wiedemann syndrome showing overgrowth and gain-of-function mutations in the PCNA-binding domain lead to SRS and IMAGe syndrome presenting with growth failure [23, 33, 34]. Only one gain-of-function mutation in *CDKN1C* was reported in SRS patients until now [33, 34]. The novel *CDKN1C* variant found in the present study (Arg316Gln) was located in the last amino acid (C-terminal) in the protein, and we showed that the amino acid substitution causes increased protein expression in vitro. Considering the location of the variant, it is likely that the variant increases the amount of protein without changing the native biochemical function of the protein. Therefore, we speculate that the increased CDKN1C protein function (i.e., gain-of-function *CDKN1C* mutation) caused the disease phenotype in patient 3. According to the result of the functional analysis, this variant was classified as “likely pathogenic” [11]. Both patient 3 and the previously reported patient did not show body asymmetry [33, 34]. The absence of body asymmetry is reasonable because *CDKN1C* mutations derive from the germline, unlike mosaic distribution of 11p15 LOM leading to body asymmetry in many patients [1]. The mother of patient 3 showing normal height had the same variant. This variant is most likely on her paternal allele.

We identified the third pathogenic *PLAG1* variant in patient 4. Both our patient and previously reported patients with *PLAG1* mutations did not show body asymmetry [3]. Our patient showed relative macrocephaly; however, two out of three previously reported patients did not [3]. In addition, patient 4 developed ADHD and hypothyroidism, but these clinical findings were not described in the previously reported patients [3]. We also tried to compare the severity of growth failure of the patients with pathogenic *PLAG1* variants with that of the patients with other (epi)genetic causes of SRS, namely, 11p15 LOM, upd(7)mat, and *IGF2* and *HMGA2* mutations. SDS of birth length in patient 4 with a pathogenic variant in *PLAG1* was − 3.12, which was comparable with those in the three previously reported patients with *PLAG1* mutations (− 2.3, − 2, and − 2.78) [3]. At birth, the lengths of the three patients with *HMGA2* mutations in the literature were − 1.3, − 3.9, and − 4.8 SDS, respectively [3, 4]. Birth lengths of previously reported patents with 11p15 LOM, upd(7)mat, and *IGF2* mutations were − 4.13 ± 2.01, − 3.18 ± 1.16, and − 4.2 ± 0.9 SDS, respectively [2, 12]. Patients with pathogenic *PLAG1* variants and upd(7)mat may show milder growth failure than those with 11p15 LOM and *IGF2* and *HMGA2* mutations. Further accumulation of patients will clarify the clinical features of SRS caused by *PLAG1* variants.

We diagnosed the genetic causes of patients 5, 6, and 7 as *IGF1R* abnormality, SHORT syndrome, and Floating-Harbor syndrome, respectively. *IGF1R* abnormality, SHORT syndrome, and Floating-Harbor syndrome are differential diagnoses of SRS because of the shared phenotypes among these syndromes and SRS [1]. Because patients 5 and 6 did not have relative microcephaly and lipoatrophy/hyperextensibility of joints, respectively, which are characteristic clinical features of *IGF1R* abnormality and SHORT syndrome [24, 28], clinical diagnosis of those in both patients might be difficult. In addition, when patient 6 was referred to us at 2 years of age, his characteristic features of SHORT syndrome were not yet detectable. The medical conditions of infantile patients or patients without typical clinical features of a specific genetic disease may be misdiagnosed as SRS by attending physicians. Two additional matters are also worth pointing out. First, the father of patient 5 who had the same *IGF1R* frameshift variant did not show severe short stature. This may be because of phenotypic variability in the patients with *IGF1R* abnormality [28]. To our knowledge, four patients with pathogenic or likely pathogenic heterozygous variants in *IGF1R* were reported to have inherited them from parents with normal stature [28, 35]. Second, patient 7 with Floating-Harbor syndrome suffered from Perthes disease. Recently, Milani et al. reported a patient with Floating-Harbor syndrome complicated by Perthes disease [36]. Our patient also developed hematoma in the left temporal lobe. Three Floating-Harbor syndrome patients with intracranial hemorrhage with a background of cerebral aneurysm have been reported [37], although our patient did not show aneurysm. Perthes disease and intracranial hemorrhage may be characteristic features of Floating-Harbor syndrome.

Patients 8 and 9 had genetically diagnosed Pitt-Hopkins syndrome and Noonan syndrome, respectively. In general, the clinical features of both Pitt-Hopkins syndrome and Noonan syndrome are not very similar to those of SRS [26, 30]. Especially, Pitt-Hopkins syndrome is not a differential diagnosis for SRS at all [26]. Patient 8 showed atypical features of Pitt-Hopkins syndrome, such as SGA and mild protruding forehead, and typical features of this syndrome, such as severe developmental delay, but not breathing abnormalities, when she was referred to us [26]. Because patients with 11p15 LOM and upd(7)mat infrequently show severe developmental delay [38], this symptom suggests other etiologies. The phenotype of patient 9 was not typical of Noonan syndrome. Noonan syndrome has variable clinical expressivity [30]. A single patient with a *PTPN11* mutation clinically suspected as SRS was reported [39]. Atypical clinical features, in some monogenic disorders, may result in clinical suspicion for SRS by presenting physicians.

Our sequencing analysis was performed in the context of an exploratory study using a multigene panel including four genes responsible for SRS and 406 genes related to growth failure and/or skeletal dysplasia. Interpreting the results of a sequencing analysis is easier using a multigene panel compared to whole-exome sequencing (WES) since the former involves a smaller number of detected rare variants. With regard to the screening of SRS phenotypic patients using a multigene panel, the genes included in the panel should focus on the genes responsible for SRS and the causative genes for genetic syndromes with clinical features that overlap with those of SRS to simplify the assessment of the results.

Our results confirmed the importance of relative macrocephaly and protruding forehead among NH-CSS criteria due to the high prevalence of “clinical SRS” in four patients with variants in the responsible genes for SRS (*IGF2*, *CDKN1C*, and *PLAG1*) and the low prevalence of “clinical SRS” in five patients with variants in causative genes for known genetic syndromes. For etiology-unknown patients who are not “clinical SRS,” genetic disorders other than SRS may be considered.

In this study, multigene sequencing showed various pathogenic or likely pathogenic variants in SRS phenotypic patients with unknown etiology. Disease-specific medical management and genetic counseling based on a precise genetic diagnosis can be used to improve the prognosis and quality of life of these patients. For example, GH therapy should be performed carefully in patient 5 with *IGF1R* abnormality and patient 6 with SHORT syndrome, due to insulin resistance and a high risk of developing diabetes mellitus [24, 28]. Similarly, a regular cardiac follow-up to screen for hypertrophic cardiomyopathy should be carried out in patient 9 with Noonan syndrome [30].

## Conclusions

We identified nine patients (9.8%) with pathogenic or likely pathogenic variants in 92 etiology-unknown patients with SRS phenotype. Notably, we identified the second *CDKN1C* and the third *PLAG1* variants leading to SRS. As a result, this study expands the molecular spectrum of SRS phenotype.

## Methods

### Patients

We included 92 patients out of 336 patients referred to us for genetic testing for SRS from 2002 to 2018 in this study. The inclusion criteria of this study are summarized in Fig. 1. For 92 patients with SRS phenotype, we ruled out 11p15 LOM and upd(7)mat by methylation analysis. None of the patients showed abnormal methylation levels for six DMRs, namely, *PLAGL1*:alt-TSS-DMR on chromosome 6, *KCNQ1OT1*:TSS-DMR on chromosome 11, *MEG3/DLK1*:IG-DMR on chromosome 14, *MEG3*:TSS-DMR on chromosome 14, *SNURF*:TSS*-*DMR on chromosome 15, and *GNAS A/B*:TSS-DMR on chromosome 20, or PCNVs. Furthermore, we excluded upd(16)mat by methylation analysis. Methylation analysis was performed by combined bisulfite restriction analysis or pyrosequencing and copy number analysis was performed using the SurePrint G3 Human CGH Array Kit 8x60K (catalog number G4450A, Agilent Technologies, Palo Alto, CA, USA) as previously reported [2,5, 40]. Our 92 patients were all Japanese, apart from two patients from Canada and the USA.

Clinical information of the patients was collected from attending physicians by questionnaire. Attending physicians consisted of general pediatricians, neonatologists, pediatric endocrinologists, and pediatric geneticists. Of the 92 patients, 63 patients satisfied NH-CSS. The remaining 29 patients met only three NH-CSS criteria but were clinically suspected as having SRS. Because triangular face, fifth finger clinodactyly, and/or brachydactyly were frequently observed in SRS patients [2] and patients are often considered as having SRS based on these features by their attending physicians, we regarded these features as clinical findings related to SRS. For patients under 23 months old, the score for postnatal growth retardation was excluded from the NH-CSS criteria.

### Molecular analysis

Using next-generation sequencing, we performed mutation screening for four genes responsible for SRS and 406 genes related to growth failure and/or skeletal dysplasia (Additional file 1: Table S1). We selected these 406 genes based on the previous reports by Meyer et al. [31] and Wang et al. [39]. Out of 92 patients, four patients were analyzed by WES and the remaining 88 patients were screened using target resequencing. For WES, enriched libraries generated using SureSelect Human All Exon V6 kit (Agilent Technologies, Santa Clara, CA, USA) were sequenced on a Hiseq X or Novaseq 6000 (Illumina, San Diego, CA, USA) operated in a 150-bp paired-end mode. For target resequencing, enriched libraries generated using a custom-made HaloPlex Target Enrichment System (Agilent Technologies, Santa Clara, CA, USA) were sequenced on a Hiseq 1500/2500/X (Illumina, San Diego, CA, USA) operated in a 100-bp paired-end mode. For WES and target resequencing, x20 coverage on average was reached in 98.7% and 89.2% of regions of interest, respectively.

Sequence reads were processed, mapped, and analyzed as previously reported [41]. In brief, adaptor sequences were removed using cutadapt 1.7.1/1.14. Sequence reads were mapped against the human reference genome data (hg19/GRCh37) using the Burrows-Wheeler Aligner 0.7.12/0.7.13. The PCR duplicates were removed by Picard 1.130/2.1.1. The Genome Analysis Toolkit 3.3/3.5 was used to perform local realignment, base quality score recalibration, and variant calling. Subsequently, we extracted rare variants based on the Genome Aggregation Database [13], Human Genetic Variation Database [14], 4.7KJPN [15], and in-house control data. Conformation and segregation of the rare variants were performed by Sanger sequencing using a standard technique. The primer sequences and experimental conditions are available on request.

Because *PLAG1* is a new responsible gene for SRS, this gene was not included in the target genes of our custom-made HaloPlex Target Enrichment System. For the 88 patients subjected to target resequencing, we performed Sanger sequencing for the coding regions and splice sites of *PLAG1* (NM_002655.3) following long PCR amplification using KOD FX Neo (Toyobo, Osaka, Japan) according to the manufacturer’s instructions. The primer sets used for Sanger sequencing are shown in Additional file 4: Table S3.

The rare variants detected by sequencing were evaluated on the bases of the American College of Medical Genetics Standards and Guidelines [11]. We extracted the variants classified as “pathogenic” or “likely pathogenic.” For in silico pathogenicity prediction, we adopted CADD [16], MutationTaster [17], SIFT [18], PolyPhen-2 (HumVar) [19], and M-CAP [20].

### Functional analysis

We established HEK293 cell lines that stably express N-terminal 3xFLAG-tagged human *CDKN1C* cDNA (WT or Arg316Gln) in the presence of doxycycline. The doxycycline-inducible piggyBac backbone vector has been described previously [42]. We introduced the human *CDKN1C* cDNA sequence (WT or Arg316Gln) into the backbone vector with the Gibson assembly technique. HEK293 cells were co-transfected with each piggyBac vector and the piggyBac transposase expression vector (System Biosciences, Mountain View, CA, USA) using Lipofectamine 3000 (Thermo Fisher Scientific, Waltham, MA, USA). Stable cells were established according to the manufacturer’s protocol.

Whole cell lysates were prepared from inducible stable cells maintained with or without 1 μg/mL doxycycline for 48 h. We performed Western blotting with anti-FLAG M2 antibody (Sigma-Aldrich, St Louis, MO, USA) and anti-tubulin antibody (Abcam, Cambridge, UK) as primary antibodies.

## Supplementary information

**Additional file 1: Table S1.** File format: Excel spreadsheet. Gene list screened in this study.

**Additional file 2: Figure S1.** File format: PowerPoint. Chromatograms of identified pathogenic or likely pathogenic variants. Arrows indicate mutated nucleotides.

**Additional file 3: Table S2.** File format: Excel spreadsheet. Summary of multigene screening studies for etiology-unknown patients with SRS phenotype using next-generation sequencing

**Additional file 4: Table S3.** File format: Excel spreadsheet. Primers utilized to detect mutations in *PLAG1*

## Data Availability

Not applicable.

## Abbreviations

ADHD: Attention-deficit hyperactivity disorder
DMR: Differentially methylated region
11p15 LOM: Loss of methylation on chromosome 11p15
GH: Growth hormone
NH-CSS: Netchine-Harbison clinical scoring system
PCNV: Pathogenic copy number variation
SDS: Standard deviation score
SGA: Small for gestational age
SRS: Silver-Russell syndrome
upd(7)mat: Maternal uniparental disomy of chromosome 7
upd(16)mat: Maternal uniparental disomy of chromosome 16
WES: Whole-exome sequencing
WT: Wildtype
