# Supplementary material for: Contribution of gene mutations to Silver-Russell syndrome phenotype: multigene sequencing analysis in 92 etiology-unknown patients
Source: Clin Epigenetics. 2020 Jun 16;12:86. doi: 10.1186/s13148-020-00865-x (PMC7298762; doi:10.1186/s13148-020-00865-x)
Supplement: Supplementary file 2 — Additional file 2: Figure S1. File format: PowerPoint. Chromatograms of identified pathogenic or likely pathogenic variants. Arrows indicate mutated nucleotides. [file 13148_2020_865_MOESM2_ESM.pptx]

## Slide 1
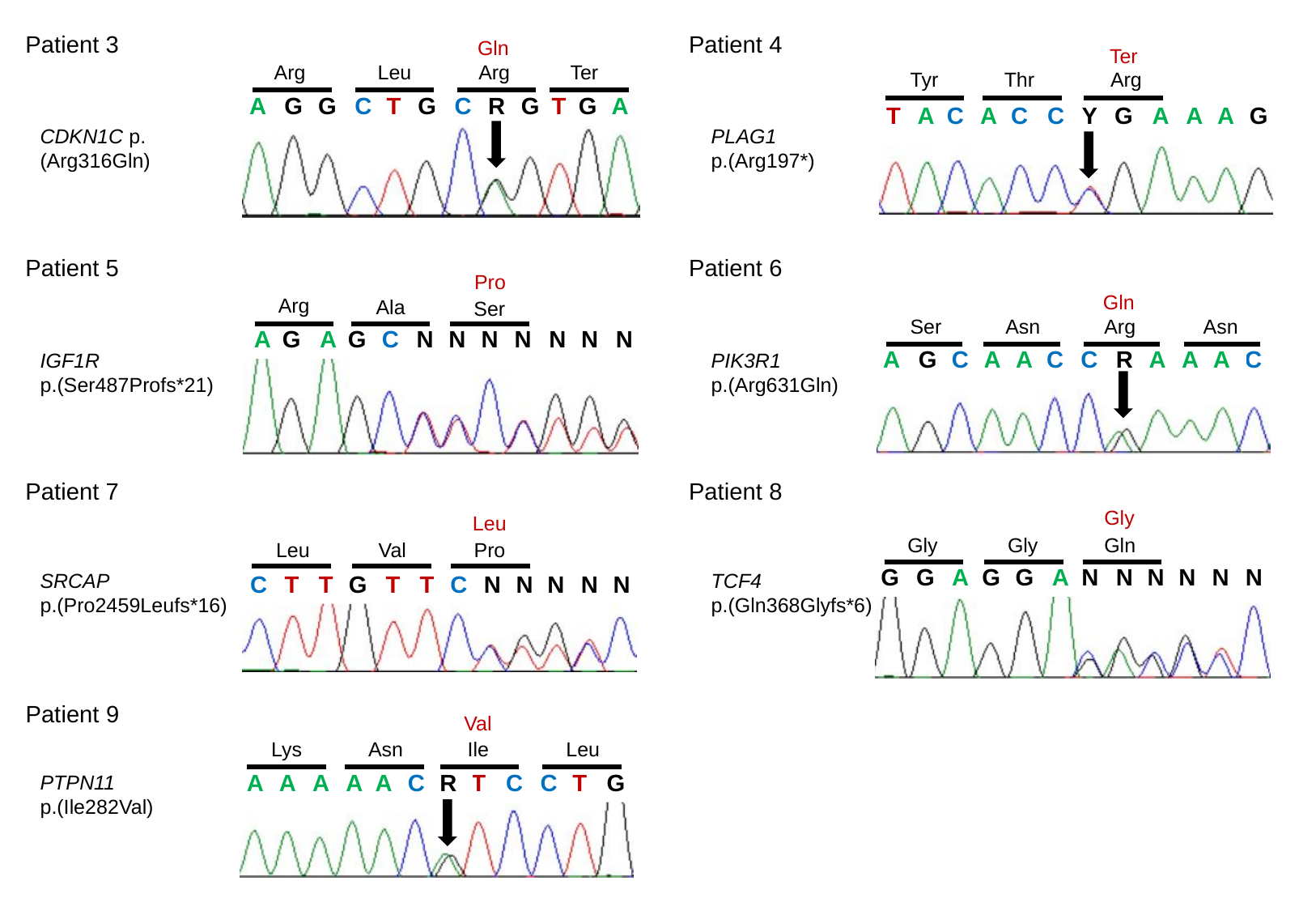

Patient 3
Patient 4
Gln
Ter
Arg
Leu
Arg
Ter
Tyr
Thr
Arg
A
G
G
C
T
G
C
R
G
T
G
A
T
A
C
A
C
C
Y
G
A
A
A
G
CDKN1C p.(Arg316Gln)
PLAG1
p.(Arg197*)
Patient 5
Patient 6
Pro
Gln
Arg
Ala
Ser
Ser
Asn
Arg
Asn
A
G
A
G
C
N
N
N
N
N
N
N
A
G
C
A
A
C
C
R
A
A
A
C
IGF1R
p.(Ser487Profs*21)
PIK3R1
p.(Arg631Gln)
Patient 7
Patient 8
Gly
Leu
Gly
Gly
Gln
Leu
Val
Pro
G
G
A
G
G
A
N
N
N
N
N
N
SRCAP
p.(Pro2459Leufs*16)
TCF4
p.(Gln368Glyfs*6)
C
T
T
G
T
T
C
N
N
N
N
N
Patient 9
Val
Lys
Asn
Ile
Leu
A
A
A
A
A
C
R
T
C
C
T
G
PTPN11
p.(Ile282Val)
